# Pressurized Hot Ethanol Extraction of Carotenoids from Carrot By-Products 

**DOI:** 10.3390/molecules17021809

**Published:** 2012-02-10

**Authors:** Arwa Mustafa, Leire Mijangos Trevino, Charlotta Turner

**Affiliations:** Department of Chemistry, Centre for Analysis and Synthesis, Lund University, P.O. Box 124, SE-221 00 Lund, Sweden

**Keywords:** accelerated solvent extraction, pressurized fluid extraction, ethanol, carrots, β-carotene

## Abstract

Carotenoids are known for their antioxidant activity and health promoting effects. One of the richest sources of carotenoids are carrots. However, about 25% of the annual production is regarded as by-products due to strict market policies. The aim of this study was to extract carotenoids from those by-products. Conventional carotenoid extraction methods require the use of organic solvents, which are costly, environmentally hazardous, and require expensive disposal procedures. Pressurized liquid extraction (PLE) utilizes conventional solvents at elevated temperatures and pressure, and it requires less solvent and shorter extraction times. The extraction solvent of choice in this study was ethanol, which is a solvent generally recognized as safe (GRAS). The extraction procedure was optimized by varying the extraction time (2–10 min) and the temperature (60–180 °C). β-Carotene was used as an indicator for carotenoids content in the carrots. The results showed that time and temperatures of extraction have significant effect on the yield of carotenoids. Increasing the flush volume during extraction did not improve the extractability of carotenoids, indicating that the extraction method was mainly desorption/diffusion controlled. Use of a dispersing agent that absorbs the moisture content was important for the efficiency of extraction. Analysing the content of β-carotene at the different length of extraction cycles showed that about 80% was recovered after around 20 min of extraction.

## 1. Introduction

Nutraceuticals and functional food ingredients are known to improve individual health with especial emphasis on elderly health since their bioactive components are reported to ameliorate or even prevent age related diseases [1,2]. Carotenoids are known for their antioxidant activity and therefore have a neuroprotective effect. They are known to have effect against singlet oxygen, inhibit LDL cholesterol oxidation, control the risk of a range of different cancers and improve cognitive development [1]. One of the richest sources of carotenoids is carrots; the reported values are in the range of 16–38 mg/100 g [3,4,5]. Fresh carrots sold in the market are subjected to strict market polices, *i.e.*, carrots should meet set standards of size and shape, and as a result of this, some 25% of the carrots produced never make it to the market. The uncontrollable nature of carrot production results in a quarter of the harvest to be regarded as by-product [6].

Pressurized fluid extraction (PFE) utilizes conventional solvents at controlled temperatures and pressure, it requires less solvent and shorter extraction times, and the set up of the technique maintains samples in an oxygen and light-free environment, which makes it preferable for use in the nutraceutical industry [7,8,9]. The advantage of applying pressure is not only that temperatures above the atmospheric boiling point of the solvents can be used, but also that it forces the extracting solvent into the matrix and therefore may improve the extraction efficiency [10]. Using pressure reduces the solvent surface tension, which facilitates the penetration of solvent into the matrix pores. It results in matrix disruption and therefore enhances the mass transfer of the analyte from the sample to the solvent. In summary, PFE improves and therefore results in efficient extraction processes. The extraction of carotenoids from different food matrices has recently been reviewed in [11]. In this paper, we have used PFE for its reported merits. 

The aim of this study was to make beneficial use of carrot by-product by extracting high-value carotenoids from them. Established methods for the extraction of carotenoids require the use of organic solvents that include *n*-hexane, propanol, methanol, tetrahydrofuran or ethyl acetate [12,13,14]. Liquid-liquid extraction of carotenoids using organic solvents is reviewed in [15]. These organic solvents are regarded as costly, environmentally hazardous, and require expensive disposal procedures. There are however several studies pointing at the use of supercritical carbon dioxide as extraction solvent for carotenoids from carrots [5,7,15]. In this study, ethanol at elevated temperature and pressure is, to the best of our knowledge, employed for the first time to create carotenoids-rich extracts from carrot by-product. Ethanol has a relatively low environmental impact and has a positive net energy balance (NEB) [16,17] and is a generally recognized as safe (GRAS) solvent. Nevertheless, the sustainability of ethanol depends majorly on the source it was produce from. 

## 2. Results and Discussion

The general aim of this study is to determine the factors that affect the extraction of carotenoids in carrot by-products using pressurized fluid technology. The different experiments were designed to be able to understand the effect of the different parameters on the extraction efficiency of carotenoids. 

### 2.1. Effect of Time and Temperature on the Extraction Yield

The content of α,β-carotenes were evaluated in two types of frozen carrot by-products, soft soggy carrots and orange carrots. The amounts of β-carotenes obtained were in the range of 12.3–22.9 mg/100 g and 8.1–19.3 mg/100 g fresh weight (FW), respectively (Table 1).

**Table 1:** Content α,β-carotenes in soft “soggy” and “orange carrots” extracted at different times and temperatures.

Temp. (°C)	No of 2-min cycles	“Soggy” carrots		“orange carrots” carrots	
		α-carotene mg/100 g *	β-carotene mg/100 g *	α-carotene mg/100 g *	β-carotene mg/100 g *
60	5	4.1	22.2	4.1	19.3
60	5	4.1	22.9	4.2	18.2
60	1	2.7	13.9	2.5	10.7
60	1	2.7	14.4	3.3	14.8
180	1	2.8	12.3	2.2	8.1
180	1	3.7	14.1	2.6	9.8
120	3	3.0	16.6	2.7	11.8
120	3	3.0	15.8	2.9	12.4
180	5	3.1	13.7	2.8	11.3
180	5	3.0	12.9	2.6	9.2

* Results are expressed in FW basis.

**Figure 1:**
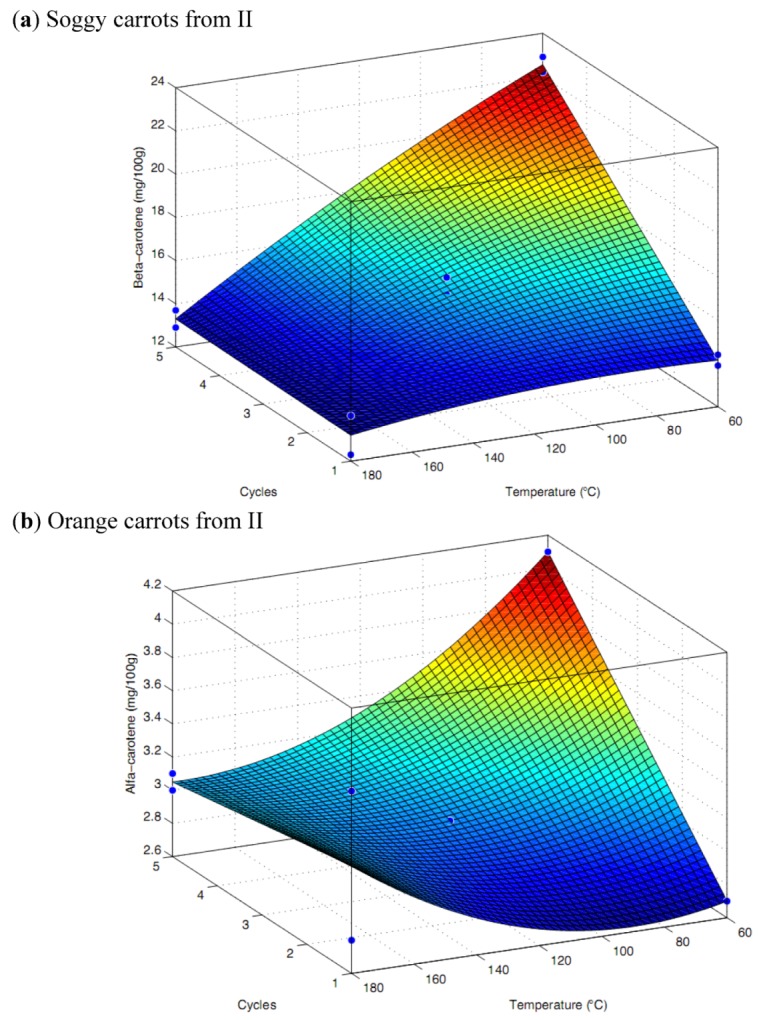
Surface plot showing the effect of time and temperature of extraction on the yield of α and β-carotene (mg/100g FW). 1 cycle = 2 min extraction.

In general, the concentration of β-carotenes was lower in orange carrots compared to the soggy carrots. The content of α-carotenes in the soggy carrots was in the range of 2.7–4.1 mg/100 g and in the fresh carrots it was in the range of 2.2–4.2 mg/100 g carrots. The α-carotene extraction yield from soggy carrots was significantly affected by time and temperature of extraction and the interaction between the two factors. The response surface design shows that both time and temperature and the interaction between these two factors significantly affect the extraction yield of α,β-carotenes, giving *p*-values of 0.002 and 0.006, respectively (Table 2). The model has adjusted R-square of 0.962, which means that the model explains about 96% of the variation. The highest yield was obtained using extraction conditions of 60 °C and 10 min extraction time (5 cycles of 2 min each) (Figure 1a,b). From the surface response design in Figure 1, it could be seen that at higher temperatures and longer times the content of α,β-carotenes were low, which could be explained by the degradation of carotenoids that are known to be relatively sensitive compounds. It has been reported that the loss of carotenes is partially due to isomerization; however oxidative degradation is the most predominant reaction [14]. It was reported in previous studies that the loss of carotenoids due to heat treatment is in the order of 10–30% [3]. Our results for β-carotene content in carrots (18–23 mg/100 g) matches previous results showing contents of β-carotene in industrial varieties that were in the range of 19–24 mg/100 g [3]. The optimal experimental conditions for extraction of carotenoids from carrots were, within the range of investigated factors, the use of ethanol at 60 °C, 50 bars, 5 min pre-heating plus 10 min extraction (5 × 2 min). Using this method, around 20 mL of 99% ethanol was used per 2.8 g of the starting material. 

**Table 2:** *P*-values for the effect of time and temperature of extraction and their interaction on the yield α,β-carotenes from response surface regression.

Factors	β-carotene	α-carotene
	*p*-values	
Time	0.004	<0.001
Temperature	0.002	0.001
Time x Temperature	0.006	0.002
R^2 a^ (%)	89.5	94.5

^a^ explained variance by the model are given as R^2^%.

**Figure 2:**
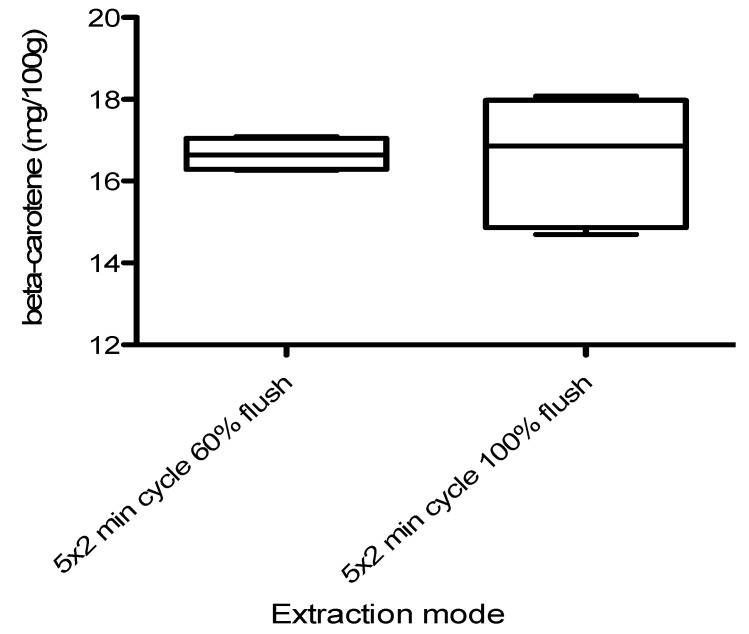
The variation in the yield of carotenoids at different times of extraction and flush per cent. Carrots type I (obtained as fresh), n = 3.

In PFE using a Dionex ASE-200**^®^**, the volume of fresh solvent introduced into the extraction cell at the beginning of each extraction cycle has varied between 16–22 mL when using flush volume of 60% and 100%, respectively. The aim of this test was to show how much content of the extractable carotenoids varies with the flush volume. Results show that increasing the amount of flush from 60% to 100% at each extraction cycle under the same extraction conditions (10 min and 60 °C) did not have a significant effect on the yield of carotenoids (Figure 2). This could indicate that the nature of the extraction at this stage is desorption/diffusion controlled, rather than solubility controlled.

### 2.2. Effect of Dispersing Agent on the Carotenoid Yield

The aim of this test was to determine if, and to what extent, the addition of dispersing or drying agent might affect the yield of carotenoids. It is crucial to know if the usage of Hydromatrix substantially improves the yield of carotenoids since its usage directly affects the economy and it also has environmental effects, especially in a larger-scale process. Nevertheless, the use of drying agent is of importance in the extraction process since the presence of water hinders the extraction efficiency. The main observation in these experiments is that in order to dry the sample it was enough to use 1 g of Hydromatrix as compared to 2.8 g in the previous experiments. This was probably due to the fact that those carrots were freshly used and not frozen and thawed as in the previous experiments. Comparing to the control runs in which no dispersing agent was used, Hydromatrix improved the extraction of β-carotene slightly, while when glass beads were used, the efficiency of the extraction was nearly unaffected (Table 3). One way ANOVA test for the different treatments showed that there was no statistical difference between the yield in the different treatments, *p*-value = 0.053. This might indicate that using dispersing agent in fresh carrot samples does not significantly improve the yield of carotenoids. The same conclusion cannot be drawn regarding freeze-stored samples since upon thawing more water would be released that might affect the extraction of carotenoids. Larger amounts of Hydromatrix would then be needed to create a dry free-flowing powder, as discussed above.

**Table 3:** Effect of using different types of dispersion/drying agents on the yield of β-carotenes in fresh orange carrots. Samples were extracted with ethanol at 60 °C for 5 times 2 min (10 min in total).

Dispersion/drying agent	Average (mg/100 g)	STDEV
Control (no dispersion)	10,3	1,4
Hydromatrix	11,9	0,9
Glass-beads	8,9	1,0

Results are presented in fresh weight basis. “n = 3”.

### 2.3. Extraction Yield during the Cycles of Extraction

**Figure 3:**
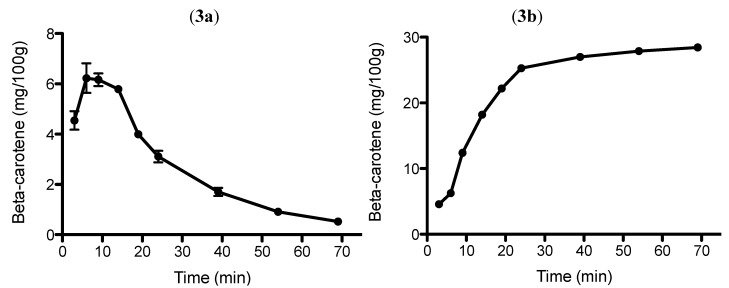
Extraction yield of β-carotenes from fresh carrots as a function of extraction time; (**A**) yield after each cycle (3 × 2 min plus 3 × 5 min plus 3 × 15 min); (**B**) extraction curve showing cumulative concentrations. Bars shows variation in replicates, n = 3. Samples were extracted at 60 °C.

The total yield of β-carotene after nine sets of extraction cycles (3 × 2 min plus 3 × 5 min plus 3 × 15 min) at 60 °C is ranging between 0.5–6.2 mg/100 g, based on average values of fresh carrots. The aim of this test was to show how much carotenoids are extractable in each set of extraction cycles and from the extraction curve it is possible to assess the extraction yield as a function of extraction time. It is also feasible to assess when the extraction becomes mainly diffusion controlled with the assumption that the extraction is solubility controlled to start with (Figure 3a). The figure shows that 78% of the total extractable β-carotene was obtained within 19 min and a total of 89% was obtained at 24 min. It is shown that the yield of carotenoids starts to level out after 24 min of extraction, after which the extraction is most likely mainly desorption/diffusion controlled. Figure 3b could be used as an illustration for an exhaustive extraction process for the extractable carotenoids, where in the last set of cycles less than 1 mg/100 g of fresh carrots were obtained. In this experiment, different lengths of extraction cycles were used to boost the extraction efficiency, *i.e.*, during the solubility controlled part of the extraction, the cycles were shorter, while during the final desorption/diffusion part of the extraction, longer cycles were employed. The total cumulative concentration of β-carotenes after 69 min of extraction (3 × 2 min plus 3 × 5 min plus 3 × 15 min) is in the order of 28.4 mg/100 g fresh carrots. The yield of β-carotenes in this experiment is substantially different from what has been shown in previous experiments due to the fact that in this study different types of carrots were used and the extraction was done in a different way. The total amount of ethanol used in this method is in the order of 170 mL with total concentration β-carotenes in the order of 15.5 mg/mL.

It should be realized that the optimal extraction conditions could not be regarded as entirely exhaustive, which might partly be explained by having some extractable carotenoids at much longer extraction times (Figure 3). However, a too long extraction time may cause degradation of the carotenoids if the extraction is conducted in one single step. The varying levels of carotenoid yields found in this paper can be explained by the different extraction procedures, the different carrot varieties that have been used, in addition to the unavoidable variation within a single carrot batch. However, within one set of experiments, such errors should be small.

## 3. Experimental

### 3.1. Materials and Sample Pre-Treatment

A systematic selection of different Swedish carrots source was made; from a carrot-farm (I), food processor (II) and marketable carrots purchased from local grocery stores (III) covering the whole chain from production to marketable carrots. Carrot by-products are any kind of carrots that does not meet the market standard specification and therefore does not make it to the market. The different sorts used in our study; parboiled, soft soggy carrots (II); whole unsorted orange carrots (II); whole unsorted orange carrots by-products (I); and fresh carrots (III). Frozen samples were thawed, grinded and homogenised using a food processor (MCM2054 Bosh, 240W). In addition, fresh carrots were purchased from a supermarket in Uppsala, Sweden, and used in some of the experiments. About 2 g of the grinded samples were used for the extraction. When dispersing agents were used, they were mixed together with the weighed-in samples before transferring the whole mixture to the extraction cell. Hydromatrix, an inert diatomaceous earth, was used as a dispersing medium, which was obtained from Varian, (Palo Alto, CA USA). Glass beads of size 2 mm were bought from VWR, Stockholm, Sweden. Carotenoids, mixed isomers from carrots, minimum 95% HPLC, ~2:1 β:α and β-carotene, minimum 95% HPLC were obtained from Sigma Aldrich (Steinheim, Germany).

### 3.2. Analysis

The HPLC methods used for the analysis of carotenoids were done using two different systems and methods, HPLC-DAD and HPLC-UV and they will be referred to as method A and B, respectively. The change in the systems was due to moving of the research lab from Uppsala to Lund University. Since β-carotene is the most abundant form of carotenoids in carrots (60–80%) [18,19], this isomer was used as an indicator for carotenoids content in carrot by-products.

**HPLC method A**: This method was carried out by HPLC-DAD (Dionex UltiMate 3000, Germering, Germany) using a YMC C-30 analytical column (150 × 2.1 mm id). Gradient elution was employed, using a mobile phase consisting of (A) methanol-water (90:10; v/v) and (B) MTBE-methanol-water (90:6:4; v/v/v). Chromatograms were monitored at a wavelength of 450 nm. 

**Figure 4:**
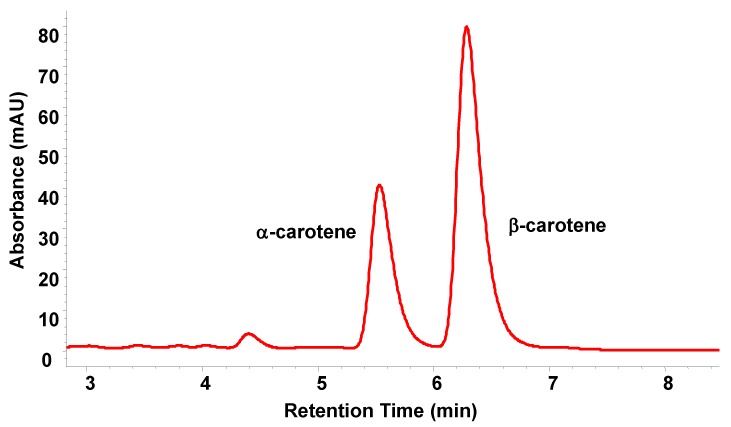
HPLC-UV chromatogram of carrot extract using PFE, λ = 450.

**HPLC method B**: This method was carried out by HPLC-UV (Agilent 1100, Waldbronn, Germany) using a YMC C-30 analytical column (150 × 2.1 mm id). Isocratic elution was employed, using a mobile phase consisting of methanol-water-MTBE 45:5:50 (vol%). Chromatograms were monitored at a wavelength of 450 nm (Figure 4). 

### 3.3. Pressurized Fluid Extraction–Commons for All Experiments

All the extraction experiments were conducted using an Accelerated Solvent Extraction (ASE-200™) equipment from Dionex Corp. (Salt Lake City, UT, USA), with the following parameters held constant: pressure (50 bars); heat-up time (5 min); flush-volume (60%); and purge time (30 s). The extraction solvent used in all experiments was ethanol (99%), degassed for 30 min by ultrasonication using a Sonorex Super 10 p, Labassco, Bandelin.

### 3.4. Pressurized Fluid Extraction-Effect of Time, Temperature and Flush Volume

The effect of varying extraction parameters on the carotenoids yield was tested by a full factorial design using carrots (II). Factors studied were time (1–5 cycles, 2 min per cycle) and temperature (60–180 °C) of the extraction. The analysis of carotenoids in these experiments was done using method A. The effect of varying the flush % on the carotenoid yield was tested by comparing the yield from the optimized conditions of time-temperature model that uses 60% flush to 100% flush using the same length of time. In this experiment the extraction temperature was kept at 60 °C and the duration per cycle was kept to 2 min. Carrots used in this test were obtained from industry B. Analysis of the carotenoids in was done using method B.

### 3.5. Pressurized Fluid Extraction-Effect of Dispersing/Drying Agent

The effect of adding dispersing agent on the extraction yield was carried out at fixed time and temperature of the extraction (60 °C and 5 cycles, 2 min per cycle) with Hydromatrix, glass-beads and no dispersing agent as a control. The yields from the different runs were compared. Carrots used in this trial were purchased fresh. Experiments were done in triplicates. The analysis of carotenoids in these experiments was done using method A.

### 3.6. Extraction Yield during the Cycles of Extraction

Extraction yield of β-carotene during the course of extraction was obtained by collecting samples after each 2-min cycle. Each cycle had a flush-volume of 100% and 30 s purge time. The first set of cycles was 3 × 2 min, the second set was 3 × 5 min and the third set was 3 × 15 min. Extracts from each set of cycle were collected in separate collection vials (nine in total) that were analyzed separately. Carrots used in this trial were obtained from I. Experiments were done in triplicate. The analysis of carotenoids in these experiments was done using method B.

## 4. Conclusions

Time and temperature of the extraction has significant effect in the yield of carotenoids. Soggy carrots have higher content of β-carotenes compared to orange carrots, while the content of α-carotenes seems to be comparable in both types of carrots. Optimized conditions for extraction are found at 60 °C, 50 bars, 5 min pre-heating plus 10 min extraction (5 × 2 min). The first stage of the extraction is a solubility controlled and the final stage was desorption/diffusion controlled. Increasing the amount of solvent does not improve the extractability of carotenoids. Using dispersing/drying agent is important of the extraction efficiency. Method presented in this study has its merits over traditional methods for been shorter in time and uses extraction solvents are regarded as "green" solvents.
